# Artificial Intelligence and Image Analysis-Assisted Diagnosis for Fibrosis Stage of Metabolic Dysfunction-Associated Steatotic Liver Disease Using Ultrasonography: A Pilot Study

**DOI:** 10.3390/diagnostics14222585

**Published:** 2024-11-18

**Authors:** Itsuki Fujii, Naoki Matsumoto, Masahiro Ogawa, Aya Konishi, Masahiro Kaneko, Yukinobu Watanabe, Ryota Masuzaki, Hirofumi Kogure, Norihiro Koizumi, Masahiko Sugitani

**Affiliations:** 1 Department of Mechanical Engineering and Intelligent Systems, Graduate School of Informatics and Engineering, The University of Electro-Communications, Chofu 182-8585, Japan; 2 Division of Gastroenterology and Hepatology, Department of Medicine, Nihon University School of Medicine, Tokyo 173-8610, Japan; ogawa.masahiro@nihon-u.ac.jp (M.O.); watanabe.yukinobu@nihon-u.ac.jp (Y.W.);; 3 Division of Pathology, Nihon University School of Medicine, Tokyo 173-8610, Japan; sugitani.masahiko@nihon-u.ac.jp

**Keywords:** ultrasonography, artificial intelligence, image analysis, liver fibrosis, fatty liver, metabolic-associated steatotic liver disease

## Abstract

Background/Objectives: Elastography increased the diagnostic accuracy of liver fibrosis. However, several challenges persist, including the widespread utilization of equipment, difficulties in measuring certain cases, and the influence of viscosity factors. A rough surface and a blunted hepatic margin have long been acknowledged as valuable characteristics indicative of hepatic fibrosis. The objective of this study was to conduct an image analysis and quantitative assessment of the contour of the sagittal section of the left lobe of the liver. Methods: Between February and October 2020, 486 consecutive outpatients underwent ultrasound examinations at our hospital. A total of 214 images were manually annotated by delineating the liver contour to create annotation images. U-Net was employed for liver segmentation, with the dataset divided into training (*n* = 128), testing (*n* = 42), and validation (*n* = 44) subsets. Additionally, 43 Metabolic Dysfunction Associated Steatotic Liver Disease (MASLD) cases with pathology data from between 2015 and 2020 were included. Segmentation was performed using the program developed in the first step. Subsequently, shape analysis was conducted using ImageJ. Results: Liver segmentation exhibited high accuracy, as indicated by Dice loss of 0.044, Intersection over Union of 0.935, and an F score of 0.966. The accuracy of the classification of the liver surface as smooth or rough via ResNet 50 was 84.6%. Image analysis showed MinFeret and Minor correlated with liver fibrosis stage (*p* = 0.046, 0.036, respectively). Sensitivity, specificity, and AUROC of Minor for ≥F3 were 0.571, 0.862, and 0.722, respectively, and F4 were 1, 0.600, and 0.825, respectively. Conclusion: Deep learning segmentation of the sagittal cross-sectional contour of the left lobe of the liver demonstrated commendable accuracy. The roughness of the liver surface was correctly judged by artificial intelligence. Image analysis showed the thickness of the left lobe inversely correlated with liver fibrosis stage.

## 1. Introduction

Metabolic dysfunction-associated steatotic liver disease (MASLD) is a prevalent condition that often leads to cirrhosis [1], liver cancer [2], cardiovascular complications [3], and other organ malignancies [3,4]. Liver fibrosis plays a critical role in the prognosis of MASLD, as it contributes to the development of cirrhosis and hepatocellular carcinoma [5], underscoring the importance of non-invasive methods for fibrosis diagnosis. The frequency of MASLD varies by region and is reported to be 25–44%. It is increasing worldwide, with an expected annual increase of 2.1% in regions with the highest rates [6].

While ultrasound elastography, which measures liver stiffness, has emerged as a method for assessing liver fibrosis [7,8], several challenges remain. These include limited access to equipment capable of such measurements [8], technical difficulties in patients with obesity [9], and the confounding effects of viscosity factors [10,11,12,13].

Elastography, available on specialized machines or high-end ultrasound systems, is not widely integrated into routine medical examinations. Viscosity factors, including inflammation [10], congestion [11], and jaundice [12], can elevate liver stiffness, complicating the interpretation of high liver stiffness as the sole indicator of advanced fibrosis.

Conversely, subjective observations from ultrasound B-mode imaging, such as irregularities in the liver surface, blunted hepatic margins [14,15], coarse parenchymal echoes [16], hepatic vein narrowing [17], splenomegaly [18], and caudate lobe enlargement [19], have long been recognized as characteristic signs of hepatic fibrosis. Furthermore, it has been established that chronic liver injury often leads to right lobe atrophy and left lobe enlargement [20], a pattern observed in two studies, including our previous study involving MASLD cases [21,22].

Our previous research revealed similar outcomes, demonstrating that a longitudinal scan of the liver’s orbit allows for the assessment of left lobe surface irregularities and blunted hepatic margins within a single image. By employing a standardized 25-image protocol for abdominal ultrasonography [23], we consistently capture and retain sagittal cross-sectional images of the left liver lobe through controlled manipulation of the pericardial region.

Recent advancements in artificial intelligence have shown promise in clinical diagnostic imaging applications. While studies have reported successes in organ identification [24], differential diagnosis of liver tumors [25], and liver fibrosis assessment using linear probes in abdominal ultrasonography [26], the subjective evaluation of liver morphology by examiners remains prevalent, with limited application of artificial intelligence in this context.

A previous study demonstrated that image analysis using the curvelet transform achieved 97.33% accuracy, 100.00% specificity, and 96.00% sensitivity in classifying ultrasound B-mode images into three categories: 50 cases of normal liver, 50 cases of fatty liver, and 50 cases of cirrhosis [27]. This study focused on quantifying parenchymal heterogeneity using cropped images of a portion of the liver parenchyma as the region of interest. However, several limitations were noted: only cases without pathological findings were included, images were not classified by fibrosis grade, and the approach may lack real-world applicability, as fatty liver and fibrosis occasionally accompanied each other.

This cross-sectional study aims to determine whether the contour of the sagittal section of the left liver lobe is smooth or rough using deep learning, and to predict fibrosis severity by employing image analysis and quantitative assessment of the contour of the sagittal section of the left liver lobe in MASLD cases.

In this study, we first utilized a dataset obtained from outpatient abdominal ultrasound scans to annotate the contours of the liver’s left lobe, enabling artificial intelligence (AI) to perform automatic segmentation. Next, two evaluators subjectively assessed the segmented images, categorizing them as either smooth or rough, and these assessments were used to train the AI. Then, AI judged as smooth or rough for test dataset. Finally, the AI-driven segmentation process was applied to a dataset of MASLD cases with pathology data, and image analyses were performed.

## 2. Patients and Methods

### 2.1. Patients and Data Collection

For machine learning analysis, 486 consecutive cases where abdominal ultrasonography was undergone in our outpatient department between February and October 2020 were utilized (Table 1). Clinical variables were obtained from hospital records for all cases. All cases in the validation and test dataset were distinct from those used in the machine learning dataset. Exclusion criteria included individuals with unclear delineation of the liver’s posterior surface, a history of hepatic resection, and those under 18 years old.

Additionally, 43 MASLD consecutive cases with pathology data between 2015 and August 2024 were included (Table 2). These specimens were obtained by liver biopsy or hepatic resection. Exclusion criteria were the same as above. Hepatitis B infection, hepatitis C infection, autoimmune hepatitis, primary biliary cholangitis, primary sclerosing cholangitis, drug-induced liver injury, Wilson’s disease, congestive liver disease, or other specific-etiology steatotic liver diseases were also excluded from these cases. Cases involving a daily alcohol intake of 30 g for males, and 20 g or more for females, were also excluded.

The pathological diagnosis was made by a pathologist who was an expert in hepatology (M.S.). The Brunt classification was used to diagnose fibrosis stage and steatosis grade [28].

The study received approval from the hospital’s ethics committee and was registered with the University Hospital Medical Information Network (UMIN) registry (UMIN000031613). It was conducted in accordance with the Declaration of Helsinki. Patients were informed about the study and had the option to opt-out.

### 2.2. Abdominal Ultrasonography

Abdominal ultrasonography was conducted with patients in a fasting, supine position. Using the pericardial fossa as the point of probe placement, a longitudinal scan capturing the periaortic area, including the hepatic border, in the inhale state, was performed systematically. The procedures were performed by a highly experienced ultrasound specialist (N.M) who was a board certificated fellow of the Japan Society of Ultrasonics in Medicine, with 22 years of expertise. The ultrasonography systems used included the Arietta 850 (Fujifilm Medical, Tokyo, Japan), LOGIQ E9, S8 (GE Healthcare, Chicago, IL, USA), and Aplio i700, 400, 300 (Canon Medical Systems, Tokyo, Japan). Convex probes were utilized, set to a depth of 14 cm, and focused at 10 cm or full focus. Image capture involved the use of Tissue Harmonic Imaging, with default settings for gain, dynamic range, and sensitivity time control (STC). Still images were saved in JPEG format, and subsequent image analysis was performed on an external PC.

Transient elastography (Fibroscan; echosens, Paris, France) with the M or XL probe being performed at the same time as the abdominal ultrasonography. Liver stiffness was determined as the median value of ten measurements, except for the minimum and maximum values. When skin–capsule distance was over 2.0 cm, XL probe was selected. Successful rates < 80% and interquartile ranges (IQR) > 40% were excluded [29].

### 2.3. Image Pre-Processing and Machine Learning for Liver Segmentation

**Figure 1:**
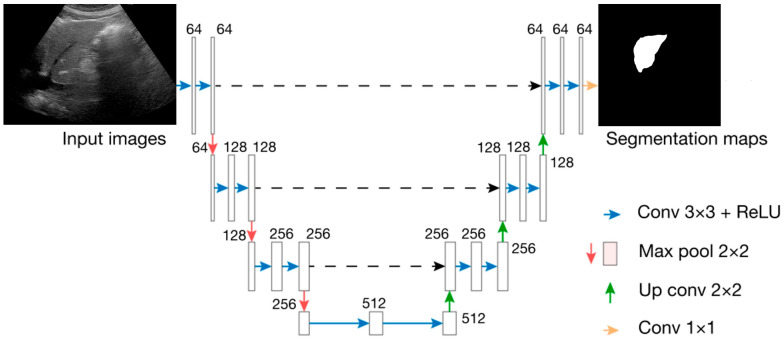
U-Net architecture.

**Figure 2:**
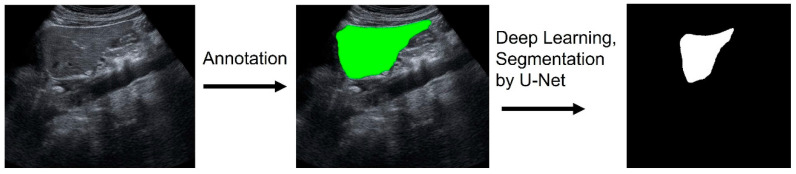
Procedure of annotation, deep learning, and automated segmentation. Annotation was performed manually. Annotation area was demonstrated as a green area. Then, the U-Net model learned these images and generated segmentation images.

**Figure 3:**
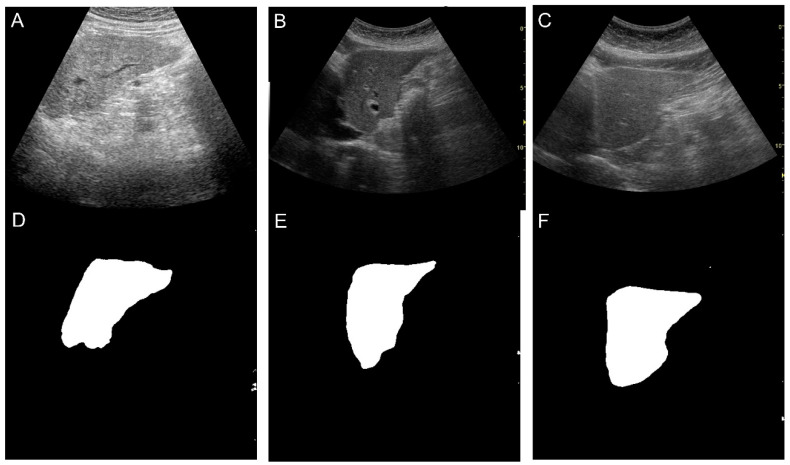
Represented cases of segmentation of the left liver lobe. (**A**–**C**) are original B-mode images. (**D**–**F**) are generated with segmentation by AI.

**Figure 4:**
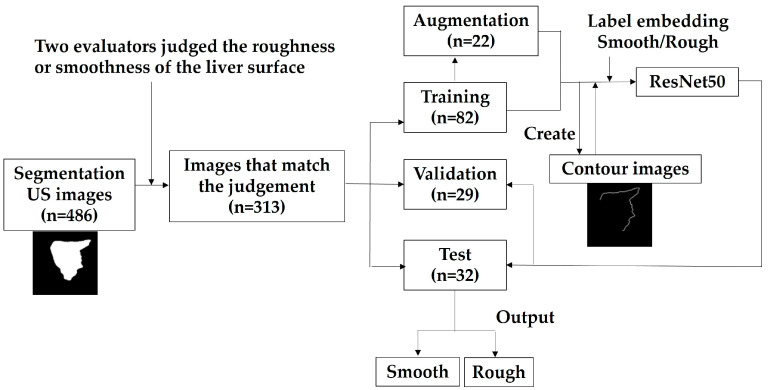
Workflow of classification via deep learning.

Out of the 486 images, 214 were manually annotated by outlining the liver’s contours by an experienced ultrasound specialist (N.M), starting from the earliest in the shooting sequence. In cases where the screen’s edge obscured the head side of the liver, those edge lines were included as part of the liver’s contour. A black box was added to the image so that the short width of the image would equal the long width, and all images were unified to be square. Initially, the U-Net model was employed for liver segmentation, using 477 cases for training [30] (Figure 1, Figure 2, Figure 3 and Figure 4).

### 2.4. Subjective Assessment

The evaluation of irregularities in the liver contour was independently performed by one ultrasound specialist (N.M.) and one image analysis specialist (I.F.). Both assessed the presence of irregularities as either present or absent. Cases with concordant results between the two were included.

### 2.5. Image Pre-Processing and Classification of the Liver Surface Roughness via Deep Learning

**Figure 5:**
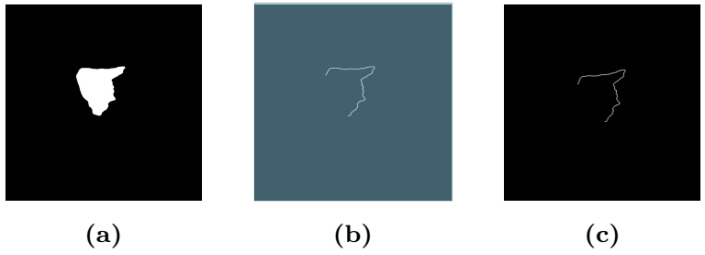
(**a**) Segmentation image. (**b**) Result after applying image process. (**c**) Results after applying affine transformation.

We propose a method to classify the roughness or smoothness of the liver surface using segmentation images generated by U-Net. For classification, we employed ResNet-50. Since the segmentation images contain information unrelated to liver fibrosis, we created images that only included the contours of the rear and front sides of the liver from the segmentation images (Figure 5). Due to the small number of images with a rough liver surface, affine transformations were applied to these images without scaling change for augmentation of the number of images. Affine transformations were performed with ±10 degree rotations and translations of 10% of the total number of pixels in the entire image for images depicting roughness. The dataset included 102 images for training, 29 for validation, and 32 for testing. Details of the dataset are shown in Table 3. We used Focal Loss [31] as the loss function, as it is robust to unbalanced datasets. The evaluation metrics included the number of images classified as rough or smooth, as well as the overall accuracy.

FL(p_t_) = −(1 − p_t_) ^γ^ log(p_t_).

### 2.6. Image Analysis

Segmentation was executed with the program established in Section 2.3, and subsequent analysis of the images was performed using ImageJ (Ver. 1.53e; National Institutes of Health, Bethesda, MD, USA).

Images were made binary; then, particle analysis was employed. The analyzed features encompassed Angle, Aspect ratio, Area, Circularity, Feret, FeretAngle, FeretX, FeretY, Height, Major, MinFeret, Minor, Perimeter, Roundness, Solidity, and Width (Figure 4); factors associated with luminance were excluded from the analysis. Image analysis was performed by a sonographer (N. M) who was blinded to the clinical data, and was a board-certified fellow of the Japan Society of Ultrasonics in Medicine (FJSUM), with 22 years of experience.

**Figure 6:**
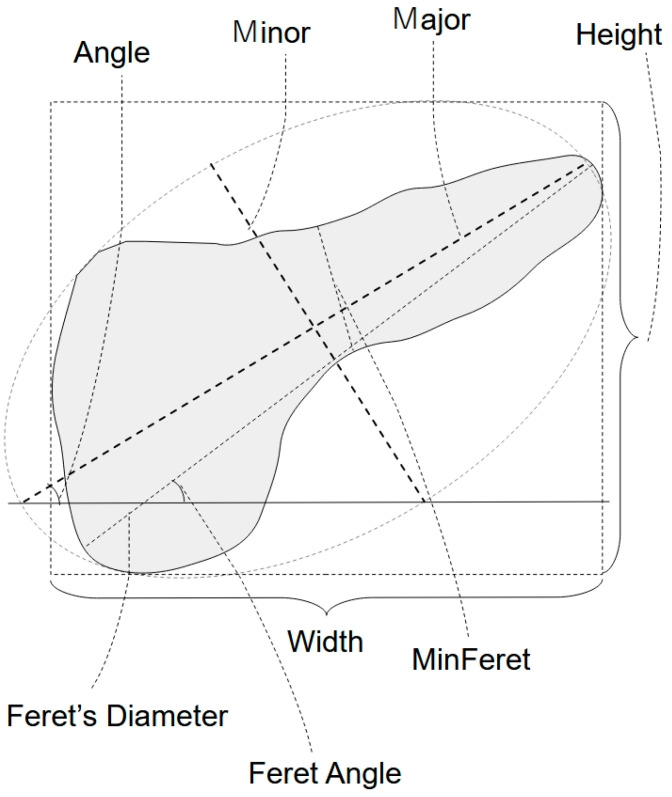
The features analyzed with ImageJ.

These parameters were automatically calculated by the ImageJ software. Definitions of each parameter are described below (Figure 6).

Area = area of selection in square pixels

Perimeter = The length of the outside boundary of the objective area

$$\mathrm{Circularity}=4\pi \times \frac{\left[\mathrm{Area}\right]}{{\left[Perimeter\right]}^{2}}$$

$$\mathrm{Solidity}=\frac{\left[\mathrm{Area}\right]}{\left[\mathrm{Convex}\cdot \mathrm{area}\right]}$$

Aspect ratio (AR) *=* major axis/minor axis

Height and Width = the height and the width of the smallest rectangle enclosing the objective area.

The following parameters are analyses of morphology about the ellipse circumscribed to the objective area.

Feret’s diameter = the longest distance between any two points along the selection boundary.

Feret angle = the angle between the Feret’s diameter and a line parallel to the *x*-axis of the image.

MinFeret = minimum caliper diameter

Major and Minor = the primary and secondary axis of the best fitting ellipse

The detailed method of this analysis was described in detail in our previous study [32]. Inter-observer agreement was confirmed in the previous report.

### 2.7. Statistical Analysis

Segmentation accuracy was assessed using Dice loss, Intersection over Union (IoU), and F-score.

Dice loss = 1 − F

$$IoU=\frac{TP}{TP+FP+FN}$$

$$F=\frac{TP}{TP+1/2(FP+FN)}$$

The Jonckheere–Terpstra test was utilized to examine the trend in the relationship between image analysis parameters and liver fibrosis. Sensitivity, specificity, and area under the curve (AUROC) were used for diagnostic accuracy in Table 4. A significance level of *p* < 0.05 was applied. EZR software, version 1.38, was used to conduct these statistical tests [33].

## 3. Results

In the Results section, we first present the accuracy of the AI in segmenting the left lobe liver contours. Next, we demonstrate the AI’s ability to distinguish between the presence and absence of contour irregularities in the left lobe of the liver. Finally, we describe the AI-based prediction of liver fibrosis through image analysis in MASLD cases.

### 3.1. Accuracy of Liver Segmentation

The results of the two evaluators’ judgments matched in 313 cases. The AI-based liver contour segmentation exhibited superior accuracy compared to manual segmentation, with a Dice loss of 0.044, an IoU of 0.935, and an F-score of 0.966.

### 3.2. Results of the Classification of the Liver Surface Roughness via Deep Learning

The results of classifying the smoothness or roughness of the liver surface of test dataset using ResNet-50 (ver.1) are shown in Table 5. Dataset 1 consists of liver contour images, while Dataset 2 includes data for the front and rear sides of the liver. “Aug.” refers to affine transformations used for data augmentation. Comparing datasets with and without data augmentation, data augmentation increased the accuracy for images with a rough surface. This indicates a reduction in the number of misclassifications. For the proposed dataset, the accuracy was over 75% for the images with roughness and the accuracy for both images with smoothness and roughness was the best score of all the datasets (Table 5). However, the methods with augmentation have lower accuracy for images with smoothness than the methods without augmentation.

### 3.3. Comparison Between Ultrasound Image Analysis Features and Fibrosis Stage

One case was excluded from the image analysis due to an unclear liver contour.

None of the items correlated with fibrosis stage, but those that did showed a correlation-included Minor (*p* = 0.049), which scored particularly poorly at F4.

**Figure 7:**
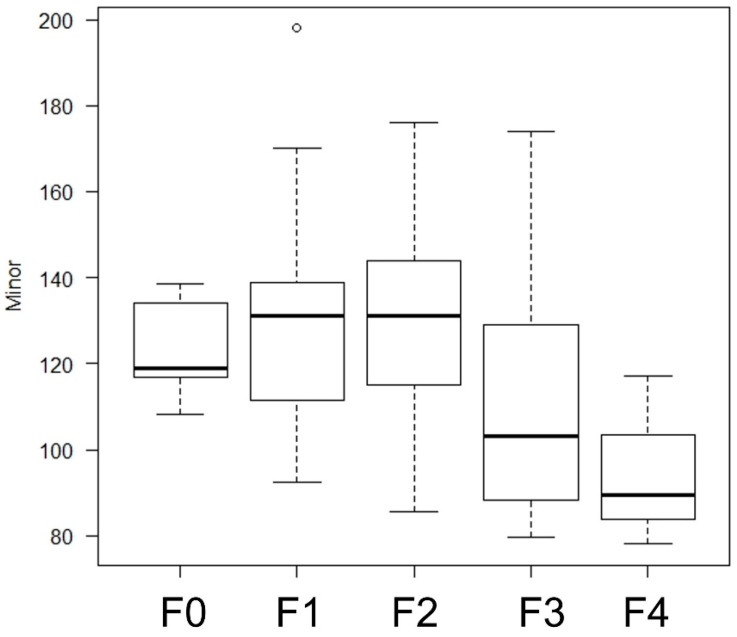
MinFeret and Minor inversely correlated with liver fibrosis stage (*p* = 0.046 and 0.036, respectively). The circle in F1 meant a maximum value at the box plot.

**Figure 8:**
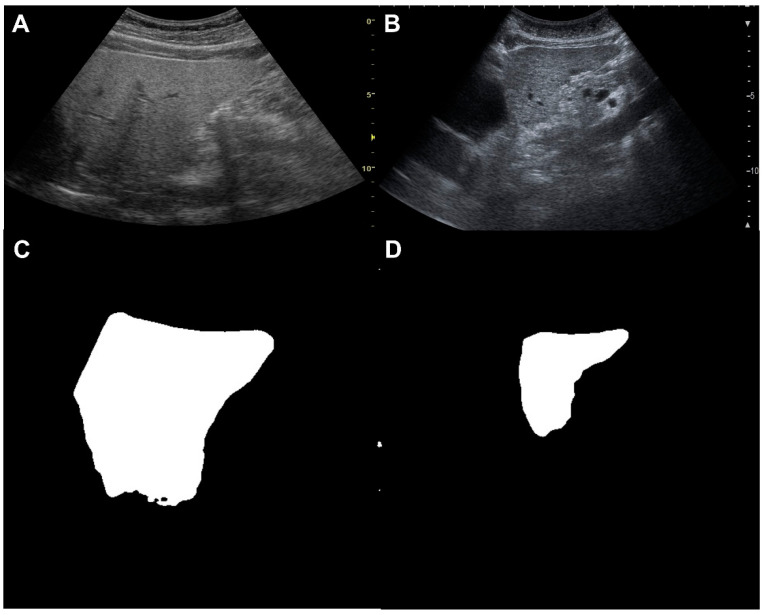
Representative cases. (**A**,**C**) are original images, and (**B**,**D**) are auto generated segmentation images. (**A**,**C**). Liver biopsy showed F1, and Minor was 198.4 in image analysis. (**B**,**D**). Liver biopsy showed F4, and Minor was 89.6 in image analysis.

Angle (*p* = 0.922), Aspect ratio (*p* = 0.190), Area (*p* = 0.060), Circularity (*p* = 0.190), Feret (*p* = 0.190), Feret Angle (*p* = 0.680), Feret X (*p* = 0.052), Feret Y (*p* = 0.269), Height (*p* = 0.124), Major (*p* = 0.209), MinFeret (*p* = 0.046), Perimeter (*p* = 0.190), Solidity (*p* = 0.657), and Width (*p* = 0.156) had little relationship with fibrosis stage (Table 6, Figure 7). Representative cases were shown in Figure 8.

Diagnostic accuracy for ≥F3 or F4 were described in Table 4. Sensitivity, specificity, F-1 Score and AUROC of Minor for ≥F3 were 0.571, 0.862, 0.615, and 0.722, respectively, and for F4 were 1, 0.600, 0.273, and 0.825, respectively.

## 4. Discussion

Our results demonstrate the excellent accuracy of ultrasound B-mode liver left-lobe sagittal section contour segmentation using deep learning. And, classification of the liver surface roughness was relatively accurate. Additionally, the smaller short-axis diameter circumscribed ellipse from the contour of the liver’s left lobe appears to be associated with the progression of liver fibrosis.

### 4.1. Application of AI in Liver B-Mode Imaging

Previous reports have classified liver imaging sections based on liver and vessel shapes. Reported liver segmentation accuracy reached an IoU of 0.8557 [34], which our study has surpassed. Moreover, many studies have reported AI-based estimation of fatty liver from images depicting the liver and right kidney [35,36]. While manual segmentation has historically been used for liver tumor assessment in B-mode images, recent studies increasingly highlight AI-driven approaches for estimating the liver fibrosis stage [37].

### 4.2. Morphological Features and Liver Fibrosis Assessment

Numerous studies have extensively explored morphological features indicative of liver fibrosis using ultrasound [14,15,16,17,18,19]. Commonly cited indicators include irregularities on the surface of the left lobe, blunt hepatic margins, coarse parenchymal echoes, hepatic vein narrowing, splenomegaly, and caudate lobe enlargement. Diagnosing liver steatosis in B-mode images presents challenges due to increased brightness and homogeneity in parenchymal echoes. However, our previous research identified irregularities on the dorsal side of the left lobe’s surface, coarse parenchymal echoes, hepatic vein narrowing, and splenomegaly in cases of hepatic fibrosis [21]. We attempted automated fibrosis diagnosis by quantifying these observations, utilizing a high-frequency linear probe for AI-based hepatic parenchymal echo diagnosis, achieving approximately 70% diagnostic accuracy [26]. The choice of the liver’s left-lobe sagittal section allowed for the assessment of surface irregularities and blunt hepatic margins. While previous studies have explored fibrosis diagnosis through AI-assessed liver parenchyma [35], including our prior work, this study represents the first report of fibrosis diagnosis via AI-assessed liver morphology.

### 4.3. Discussion of Features of Image Analysis and Liver Fibrosis

Solidity, indicating target irregularity, was initially assumed to decrease in cirrhosis. However, our results revealed no discernible association with liver fibrosis stage. Similarly, the lack of correlation observed in Circularity and Aspect ratio could be attributed to the sagittal cross-sectional liver image’s inherent dissimilarity to a circular shape, yielding lower values even in a non-fibrotic liver. Notably, our findings suggest atrophy in the left lobe, potentially indicating progressive cirrhosis. Further advancement into cirrhosis could lead to whole liver atrophy, possibly captured in our study.

### 4.4. Diagnosis of Liver Steatosis and Cirrhosis in Ultrasound Images Using Artificial Intelligence and Image Analysis

Numerous computer-assisted studies have been conducted to diagnose fatty liver from ultrasound B-mode images, as well as to differentiate between normal liver, fatty liver, and cirrhosis [27,38,39]. These studies primarily employ image analysis techniques that examine the speckle patterns of the liver parenchyma. Artificial intelligence (AI) has also been explored in this field for some time, initially through the use of neural networks and more recently through deep learning approaches [40]. One significant issue with these studies is the lack of standardized cross-sectional images, with the region of interest (ROI) often being manually selected to avoid blood vessels [27,41]. Furthermore, in AI-based studies, the reasoning behind the decision-making process is unclear, leaving it uncertain whether AI is actually analyzing the liver parenchyma’s speckle pattern. The manual selection of the ROI also introduces potential human bias.

In the present study, we aimed to eliminate such biases by implementing automatic segmentation. Analyzing the shape of the liver represents a novel approach, as, to the best of our knowledge, this perspective has not been explored in previous studies. By consistently capturing images of the same cross-sectional area of the liver, following the methodology outlined in the Manual for abdominal ultrasound in cancer screening and health checkups [23], we were able to conduct this study.

The accuracy in this study, however, was relatively not so high (Table 4). Only two parameters (MinFeret and Minor) showed a significant correlation between image analysis and pathology, in contrast to the more than 90% accuracy reported in previous studies. Earlier studies typically classified the liver into two categories (normal liver and fatty liver) or three categories (normal liver, fatty liver, and cirrhosis). Diagnosing the progression of hepatic fibrosis in fatty liver is particularly challenging due to the homogeneity of the liver parenchyma. Given the clinical importance of diagnosing fibrosis in fatty liver and our previous findings indicating that the liver’s posterior surface is uneven, we sought to apply AI and image analysis in this study to assess surface irregularities and shape alterations in the liver contour.

### 4.5. Limitations

The primary limitation of this study is the relatively small sample size for deep learning analysis. Strategies to address this, beyond merely increasing the number of sample images, such as utilizing videos or data augmentation, carry a risk of overfitting. Second, the annotation of the liver contour was performed with hand-drawing. Even if the outline was drawn very carefully, the accuracy of the annotation has limitations, because gas in the stomach or lymph node partially obscures the dorsal side of the liver. And, as annotations made by more than two individuals have more reliability, the robustness of the findings of this study are limited. Additionally, the limited number of image analysis features used may overlook other potentially useful parameters that could be crucial for fibrosis diagnosis.

### 4.6. Future Prospects

We aim to leverage the insights from our study to enhance liver fibrosis diagnosis through artificial intelligence, with a focus on sagittal section contours of the left liver lobe. The spleen size and narrowing of the hepatic vein might be useful to predict the hepatic fibrosis stage, and these data can be classified with image analysis or artificial intelligence. Then, by integrating these parameters, we believe that liver fibrosis stage can be predicted with high precision. The next step is to apply this approach to pathologically proven MASLD cases and verify its performance in predicting fibrosis stages.

## 5. Conclusions and Future Work

Deep learning-based segmentation of the sagittal cross-section contour of the left liver lobe demonstrated high accuracy. Artificial intelligence was relatively effective in judging the roughness of the liver surface. While thinner cross-sections were more likely associated with F4, the limited case number warrants further accumulation and reassessment.

We aim to leverage the insights from our study to enhance liver fibrosis diagnosis through artificial intelligence, with a focus on the sagittal section contour of the left liver lobe. The spleen size and narrowing of the hepatic vein might be useful to predict hepatic fibrosis stage, and these data can be classified with image analysis or artificial intelligence. Then, by integrating these parameters, we believe that liver fibrosis stage can be predicted with high precision. The next step is to apply this approach to pathologically proven MASLD cases and verify its performance in predicting fibrosis stages.

## Figures and Tables

**Table 1:** Patient characteristics in images used for deep learning.

N	486
Age	63.4 ± 14.1
Male/female	220/266
Skin–capsular distance [cm]	1.5 ± 0.3
Fibroscan [kPa]	7.9 ± 7.7
Etiology	
HBV	83
HCV	93
HBV + HCV	2
Alcohol	42
MASLD	52
Autoimmune hepatitis	22
Primary biliary cholangitis	28
Others	167
Condition of the liver	
Normal liver	95
Liver steatosis	82
Chronic liver damage	218
Liver cirrhosis	54
Chronic liver damage + steatosis	32
Liver cirrhosis + steatosis	3
Congestive liver	4

HBV, hepatitis B virus; HCV, hepatitis C virus; MASLD, metabolic dysfunction-associated steatotic liver disease.

**Table 2:** Patient characteristics of pathologically proven MASLD for image analysis.

N	43
Age	57 (20–79)
Male/female	22/21
Body mass index	26.6 (19.9–36.9)
AST [IU/L]	53 (24–170)
ALT [IU/L]	64 (19–398)
Platelet [mm^3^]	21.9 (8.3–37.9)
Fibrosis stage (0/1/2/3/4)	5/11/13/11/3
Steatosis grade (S0/S1/S2/S3)	0/24/15/4

AST, aspartate aminotransferase; ALT, alanine aminotransferase.

**Table 3:** Dataset to learn, validate, and test a binary classification.

	Images with Roughness	Images with Smoothness	Augmentation
Training	22 (26.8%)	60 (73.2%)	22
Validation	8 (27.6%)	21 (72.4%)	
Test	9 (28.1%)	23 (71.9%)	

**Table 4:** Diagnostic accuracy of image analysis for ≥F3 or F4.

	Diagnosis for ≥F3				Diagnosis for F4			
Variables	Sensitivity	Specificity	F-1 Score	AUROC	Sensitivity	Specificity	F-1 Score	AUROC
Aspect ratio	0.714	0.586	0.556	0.600	1	0.575	0.261	0.758
Area	0.500	0.931	0.609	0.707	0.667	0.900	0.445	0.775
Circularity	0.500	0.828	0.499	0.596	1	0.775	0.401	0.817
MinFeret	0.786	0.621	0.612	0.723	1	0.550	0.250	0.767
Minor	0.571	0.862	0.615	0.722	1	0.600	0.273	0.825
Height	0.500	0.862	0.560	0.677	1	0.600	0.273	0.762

AUROC, area under the receiver operating characteristic curve.

**Table 5:** Accuracy of the classification of the liver surface roughness via deep learning.

	Accuracy [%]		
	Image with Smoothness	Image with Roughness	Both Images with Smoothness and Roughness
Dataset 1	100.0	0.0	73.1
Dataset 1 + Aug.	63.2	100.0	73.1
Dataset 2	94.7	14.3	73.1
Dataset 2 + Aug. (Proposed Dataset)	69.5	88.9	75.0

**Table 6:** Image analysis of the sagittal section of the left lobe.

Variables	*p*
Angle	0.922
Aspect ratio	0.190
Area	0.060
Circularity	0.190
Feret	0.190
FeretAngle	0.680
FeretX	0.052
FeretY	0.269
Height	0.124
Major	0.209
MinFeret	0.046
Minor	0.036
Perimeter	0.190
Solidity	0.657
Width	0.156

## Data Availability

The raw data supporting the conclusions of this article will be made available by the authors on request.
